# Saccharine Diabetes

**Published:** 1877-11

**Authors:** 


### Selections.

Saccharine Diabetes.—A. H., aged 24, was perfectly well until 1871, when he suffered from abdominal colic. He recovered from this attack, and continued well until last December, when he began to grow weak, and to crave food and drink. His urine was greatly in excess. He continued to grow more and more emaciated. He also complained of pain in the right hypogastric region, and of shortness of breath on exertion. I can learn nothing with regard to the boy's parents. He seems to have had a very hard life of it thus far; received blows on his head very frequently when young.

The general and special symptoms of diabetis are well known. The urine is almost always very greatly in excess. The sufferer is obliged to get up frequently during the night, to pass urine. The natural quantity of urine passed in twenty-four hours is about thirty fluid ounces; in diabetes sometimes as much as ten quarts is passed in the same time. In severe cases the quantity is even greater than this—as much as three, or even four gallons during day and night. Excessive diuresis, therefore, is a marked symptom. Saccharine diabetes must be distinguished from Bright's disease or albuminuria. In Bright's disease there is albumen, and no sugar in the urine, and the specific gravity of the urine is, as a general rule, below the normal, about $\frac{1005}{1000}$. Drops and granular degeneration generally accompany the disease. In saccharine diabetes the specific gravity is always above normal—that is, above 1020, the stools are hard and dry, and sugar is always present in the urine. There may be cases of simple excessive diuresis without the presence of sugar. In the present case the urine is very light-colored, slightly turbid, and with a very high specific gravity, above 1033. This boy has passed three hundred fluid ounces of urine in twenty-four hours. His urine contains a large quantity of glucose. In diabetes you nearly always find excessive thirst and craving for food; usually there is a direct proportion between the thirst and the amount of urine passed. The weakness may be excessive before the unusual excess of urine be noticed. In this disease the skin is usually harsh and dry. There is very rarely any sweating. Sometimes the presence of sugar in the blood brings on itching and eruptions of various kinds on the skin. These eruptions are often very difficult to cure. The tongue is red, the throat irritated, and the digestion fails. In later stages it is customary to find a good deal of dyspepsia, with eructions and general torpidity of the bowels. If the case progresses to an unfavorable termination there are evidences of profound mal-nutrition, obstinate diarrhœa, slow, dry gangrene, and inability to bear any strain or shock. Surgical operations at this period are generally followed by rapid gangrene and fatal results.

The sugar found in the urine in this disease comes from the starchy foods eaten. The blood of the portal veins contains, in health, a considerable quantity of glucose, which in the normal subject is broken up into blood and carbonic acid. This is the natural healthy process which is stopped in saccharine diabetes. The starch is changed in digestion into glucose, and reaches the portal vein in that state, but never goes beyond that stage, and therefore goes into the general circulation as glucose. The liver plays a very important part in diabetes. Not only does the starchy food taken never pass beyond the stage of glucose, but it is also probable that the so-called glycogenic, or animal glycogen-producing function of the liver is unusually, abnormally, active in diabetes.

A certain set of organic diseases of the brain are attended with diabetic symptoms. These diseases all affect the floor of the fourth ventricle, near the origin of the pneumogastric nerve. Experimental lesions of the floor of this ventricle are known to cause sugar to appear in the urine.

Diabetes occurs in all ages in both sexes. All classes of persons are liable to it. There is often seen a set of *diabetics* past middle life, who manage, in spite of their disease, to keep up flesh end strenth. There is not much sugar in the urine, and the case is generally of easy management. In youth, diabetes is always attended with more or less danger.

In the hygienic treatment of this disease, the first effort must be toward the cutting off the supply of all starchy elements of food. If the urine becomes less abundant under this exclusive regimen, some hope of the ultimate cure may be held out. In some very obstinate cases, even after excluding from the food starchy matters, the urine still continues to contain a large proportion of sugar.

In all cases the diet must be modified, as above stated. Much time might profitably be spent in the consideration of the diatetics of this disease, and I shall have to postpone this until a future occasion. As regards the medicinal methods, I have derived the best of results from the continuous administration of large doses of opium; as much as ten grains may be given daily, in divided doses, without producing the slightest drowsiness. This treatment has been followed in the present case by a great decrease in the amount of urine and of sugar contained. The proportion of sugar, however, has not yet gone down. The boy is taking now five grains of opium daily, and the amount of urine has fallen from twenty to eleven pints daily. As regards the prognosis of this consuming and weakening complaint, nothing favorable can be offered, until the sugar begins to disappear from the urine.—*Phila. Med. and Surg. Reporter.*

